# Propofol Requirements for Induction of Anaesthesia in Dogs with Cervical or Thoracolumbar Myelopathy Due to Intervertebral Disc Disease

**DOI:** 10.3390/ani16131992

**Published:** 2026-06-28

**Authors:** Eirini Sarpekidou, Maria Koura, Kiriaki Pavlidou, Vasileios Zapridis, Ioannis Savvas, Zoe Polizopoulou, George Kazakos

**Affiliations:** 1 Surgery & Obstetrics Unit, Companion Animal Clinic, School of Veterinary Medicine, Faculty of Health Sciences, Aristotle University of Thessaloniki, 54627 Thessaloniki, Greece; e.sarpekidou@gmail.com; 2 Anesthesia and Intensive Care Unit, Companion Animal Clinic, School of Veterinary Medicine, Faculty of Health Sciences, Aristotle University of Thessaloniki, 54627 Thessaloniki, Greece; mariak1298eng@gmail.com (M.K.); kellypav@gmail.com (K.P.); vzaprides@gmail.com (V.Z.); isavas@vet.auth.gr (I.S.); 3 Diagnostic Laboratory, Companion Animal Clinic, School of Veterinary Medicine, Faculty of Health Sciences, Aristotle University of Thessaloniki, 54627 Thessaloniki, Greece; poliz@vet.auth.gr

**Keywords:** anaesthesia, propofol, dexmedetomidine, IVDD, cervical, cervicothoracic, thoracolumbar, dogs

## Abstract

**Background:** The aim of the present retrospective observational study is to compare the required doses of propofol (PPF) for anaesthesia induction in dogs presented with intervertebral disc disease (IVDD)-related thoracolumbar or cervical myelopathy. **Methods:** The anaesthetic records of dogs with either cervical- or thoracolumbar-related myelopathy anaesthetised for diagnostic or surgical procedures from September 2021 to July 2023 were evaluated. Inclusion criteria were premedication with the same dose of dexmedetomidine (DEX) (180 μg m^−2^), and induction with PPF. The sedation score (SS), Glasgow Composite Measure Pain Scale-Short Form (GCMPS-SF) results and the total PPF dose required for intubation were collected. Data were analysed with the Shapiro–Wilk test, independent samples *t*-test and Spearman’s rho test. A value of *p* < 0.05 was considered statistically significant. **Results:** Fifty-five records were included in the study, 27 dogs with cervical and 28 with thoracolumbar myelopathy due to IVDD. Statistically significant higher doses of PPF were required in the dogs with cervical (mean 3.2 mg kg^−1^, ±SD 1.25 mg kg^−1^) than with thoracolumbar (mean 1.9 mg kg^−1^, ±SD 0.65 mg kg^−1^) myelopathy (*t*-test, *p* < 0.001). The SS in the dogs with cervical–cervicothoracic myelopathy (CCTM) (median 6, ±min 2 max 10) also differed from the SS of the dogs with thoracolumbar myelopathy (TLM) (median 7.5, min 2 max 12) (*t*-test, *p* = 0.02). Sedation score and the dose of PPF were significantly negatively correlated (Spearman’s rho −0.311, *p* = 0.021). Sedation score and GCMPS-SF were also significantly negatively correlated (Spearman’s rho −0.363, *p* = 0.007). However, no statistically significant negative correlation was found between PPF dose and GCMPS-SF score (Spearman’s rho = 0.248, *p* = 0.068). **Conclusions:** Our findings should be considered when planning anaesthetic protocols in patients suffering from IVVD-related myelopathy in order to optimize safety in these patients.

## Simple Summary

General anaesthesia is often necessary for the diagnostic and surgical management of dogs presenting with signs of spinal cord disease due to intervertebral disc disease. Premedication is essential for a balanced anaesthetic protocol and its effect may vary, depending on the degree of excitement, pain and stress. α2 adrenergic agonists are often included in the pre-anaesthetic medication protocol in neurological patients. They have dose-sparing effects of the injectable anesthetic agent needed for intubation, depending on the level of sedation which was achieved with premedication. The aim of this study is to investigate differences in propofol requirements for anaesthesia induction in dogs that were presented with cervical or thoracolumbar myelopathy associated with intervertebral disc disease, premedicated with a standard dose of dexmedetomidine. Results suggest that dogs with cervical myelopathy have higher propofol requirements for intubation while dogs with thoracolumbar myelopathy could be intubated with lower doses. The findings of the present study should be considered when planning anesthetic protocols for these patients in order to optimize anesthetic safety.

## 1. Introduction

General anaesthesia is required for diagnostic procedures (computed tomography, magnetic resonance imaging, radiographs) or surgical intervention in dogs presenting with signs of focal compressive myelopathy associated with intervertebral disc disease IVDD [1,2]. In the absence of underlying comorbidities or severe pathological changes resulting from IVVD, patients suffering from compressive myelopathies fall into ASA (American Society of Anesthesiologists) II category [3,4]. Anaesthetic protocols recommended for ASA II patients vary in the veterinary literature. However, these protocols often include α2 agonists [5,6]; thus, dexmedetomidine (DEX) is an appropriate premedication agent for these patients [5]. Dexmedetomidine is one of the most common premedication drugs used in veterinary medicine, as it offers great and reliable sedation [5,7,8,9].

Following sedation, anesthesia induction can be achieved using various injectable anesthetics. Propofol (PPF) is probably the most popular injectable agent for anaesthesia induction as it has a rapid onset, a very quick recovery and eases intubation [10,11,12]. Propofol’s clinical effect (hypnosis) is mediated through GABA receptors; however, their interaction is yet to be completely clarified [10,11].

The level of dexmedetomidine-produced sedation will greatly affect the required doses of PPF for intubation. Dexmedetomidine-induced sedation may be reduced by high stress levels and painful pathological conditions [5,13]. Patients that suffer from spinal cord disease often exhibit signs of pain, which in some cases can be very severe. This results in stress, discomfort caused by handling and in cases of chronic neuropathic pain even allodynia [14]. This emphasizes how important a reliable premedication is in preventing PPF overdose and minimizing the need for volatile anesthesia and its many dose-dependent adverse effects [4,7].

In dogs with IVDD affecting the cervical and thoracolumbar spinal cord, the PPF dose required for induction of anesthesia has not yet been established. This is particularly important given that dogs with focal myelopathy may present with diaphragmatic impairment, as recently reported by our research group in these patients [15]. PPF administration may further compromise respiratory function because of its dose-dependent respiratory depressant effects. Propofol administration is frequently associated with apnea and reduced ventilatory response to hypoxemia and hypercapnia [16]. Therefore, the administration of PPF in dogs with respiratory compromise secondary to cervical and thoracolumbar spinal cord lesions should be carefully controlled [15].

The aim of the study is to compare the PPF dose requirements in ASA II dogs with focal compressive myelopathy in the thoracolumbar and in the cervical spinal column due to IVDD, premedicated with the same dose of DEX. We hypothesised that PPF dose requirements would differ between dogs suffering from CCTM and TM.

## 2. Materials and Methods

This retrospective analysis was performed using anesthesia record data from a prospectively collected cohort of dogs enrolled in a standardized research protocol. The study included dogs diagnosed with intervertebral disc disease (IVDD) associated with cervical–cervicothoracic myelopathy (CCTM) or thoracolumbar myelopathy (TLM) that were presented to the Anaesthesia Unit of the Companion Animal Clinic, Aristotle University of Thessaloniki, between September 2021 and July 2023. Dogs between 1 and 11 years that exhibited a body weight that was within the normal range for their breed and age were included. All dogs had neurological deficits associated with CCTM or TLM. That at our institution is defined by loss of jaw tone, absence of resistance to protraction of the tongue, and absence of the gag reflex. Only dogs characterised as ASA ≤ II were included in the study. Records were divided into two groups. Group CCTM included dogs with an IVDD spinal cord defect located within the first cervical (C) vertebra and the second thoracic (T) vertebra, and group TLM included dogs in which the spinal cord defect was within T3 and the third lumbar (L) vertebra. Only dogs that received a single dose of DEX 180 μg m^−2^ (Dextomidor 0.5 mg/mL, Orion Pharma, Espoo, Finland) intramuscularly (IM) followed 30 min later by intravenous (IV) PPF (Propofol MCT/LCT/Fresenius 1% 10 mg/mL, Fresenius Kabi, Graz, Austria), 1 mg kg^−1^, followed by additional 0.5 mg kg^−1^ boluses administered every 30 s titrated to effect until intubation criteria were met, were included. The anaesthetic procedure was performed or under the supervision of the same anesthesiologist (KP) as part of another research study. After the PPF administration, anaesthesia was maintained with isoflurane in 100% oxygen and the rest of the procedure followed based on the protocol.

Anaesthesia and medical records were reviewed by 6 authors (E.S., M.K., V.Z., K.P., G.K., and I.S.), and data collected included patient demographics (age, breed, weight, sex), procedural information, IVDD location, diagnosis (Hansen type I/II), Modified Frankel Scoring (MFS) system, pre-anaesthetic pain score, sedation score [17] using a 5-point clinical scoring system for the evaluation of sedation depth, with a total score ranging from 0 to 17. Anaesthetic drug protocols (propofol dose), and the co-administration of other medications prescribed for the clinical management of IVDD at the time of anesthesia were also assessed and categorized into drug classes.

The anesthesia records were divided into two groups ARCCTM (anesthesia record cervical–cervicothoracic) and ARTLM (anesthesia record thoracolumbar) and the doses of PPF (mg kg^−1^ of body weight) were evaluated. Apart from the anesthetic records, case files were reviewed to retrieve information that might have contributed to the amount of PPF administered. Thus, pain scoring records conducted using the Pain Scale—GCMPS-SF, pre-anesthetically, were also evaluated. Moreover, the sedation scoring [17] records that were conducted 20 min after the DEX administration and before PPF administration, were also collected and evaluated. The PPF doses required for intubation in group CCTM and TLM were assessed in relation to GCMPS-SF scoring and to SS.

These included anti-inflammatory drugs, such as corticosteroids or nonsteroidal anti-inflammatory drugs (NSAIDs), gabapentinoids (gabapentin or pregabalin), and opioids. Since tramadol was the only opioid administered, this group was designated as the tramadol group.

Dogs were excluded from the study if anaesthetic records were incomplete and sedation scores or GCMPS-SF pain scoring data were missing. Additional exclusion criteria included body weight outside the normal range for breed and age, age below 1 year or above 11 years, and an ASA classification other than ASA II. Dogs without a definitive diagnosis of IVDD-associated CCTM, or TLM were also excluded, as were those with concurrent systemic disease or comorbidities that could influence anaesthetic requirements or physiological responses. Furthermore, dogs were excluded if they received premedication or induction protocols deviating from the standardized regimen of a single intramuscular dose of dexmedetomidine (DEX 180 μg m^−2^) followed by PPF induction as described. Cases requiring additional sedative or anaesthetic agents not included in the protocol, or those presenting as emergency cases with unstable clinical status at the time of induction, were also excluded.

### Statistical Analysis

All data were evaluated for normality with the Shapiro–Wilk normality test. Normally distributed data were presented as mean ± standard deviation (SD), and non-normally distributed data as median (range). Independent samples *t*-test was used to evaluate any significant differences in the PPF dose. Spearman’s rho was used to evaluate any correlations between SS score and PPF dose, SS score and GCMPS-SF score, and PPF dose and GCMPS-SF score. Statistical significance level was set to α < 0.05. For all statistical calculations, a dedicated software was used (JASP 0.19.3).

## 3. Results

### 3.1. Demographics

In this retrospective study, a total of 55 (*n* = 55) anesthesia records were retrieved from dogs suffering from IVDD-associated CCTM (*n* = 27) or TLM (*n* = 28) between September 2021 and July 2023 (Demographics Table A1). Breeds in group CCTM were French Bulldog, Mixed Breed, Cocker Spaniel, Chihuahua, Miniature Pinscher, Greek Shepherd Dog, Labrador Retriever, Beagle and German Shepherd Dog, median age of the animals was 7 (3–10) years and mean weight was 12.5 (±SD 7) kg. There were 16 male dogs and 10 female dogs. Among the females, nine were spayed and one was intact. Among the males, nine were neutered and seven were intact.

In group TLΜ, breeds Miniature Pinscher, Maltese, Mixed Breed, Pekingese, West Highland White Terrier, French Bulldog, American Pit Bull Terrier, Jack Russell Terrier and Pug were included, median age of the animals was 6 (2–10) years and mean weight was 10,4 kg (±SD 5.1). There were 17 male dogs and 11 female dogs. Among the females, ten were spayed and one was intact. Among the males, nine were neutered and eight were intact.

All animals were premedicated with DEX 180 mg kg^−1^ (IM). After 20 min, an intravenous catheter was placed in the cephalic vein and PPF was administered at a dose of 1 mg kg^−1^ followed by 0.5 mg kg^−1^ doses (to effect) until intubation was possible.

### 3.2. Propofol Doses

Dogs suffering from CCTM required a significantly higher dose of PPF than dogs suffering from TLM (3.2 ± 1.25 mg kg^−1^ versus 1.9 ± 0.65 mg kg^−1^, respectively; *p* < 0.05) (Table A2).

### 3.3. Glasgow Composite Measure Pain Scale-Short Form

Due to the fact that many animals were non-ambulatory at the time of presentation, GCMPS-SF was assessed using either the 20-point or the 24-point scoring system, depending on the individual dog’s neurological status. To allow direct comparison between cases evaluated with different total scores, all GCMPS-SF results were standardized by converting each score into fractional values relative to the maximum achievable score (Table A2). Thus, the value of GCMPS-SF was 0.49 (0–0.75) in group CCTM and 0.31 (0–0.7) in group TLM. The differences in GCMPS-SF results were not statistically significant between the CCTM and TLM group (Table A2).

### 3.4. Sedation Score

Dogs with CCTM had a median sedation score significantly higher than dogs with TLM 6 (2–10) in CCTM versus 7.5 (2–12) in TLM (*p* = 0.02) (Table A2).

### 3.5. Propofol Dose, Glasgow Composite Measure Pain Scale-Short Form and Sedation Score Correlation Tests

There seems to be a negative correlation between SS and the dose of PPF (Spearman’s rho −0.311, *p* = 0.021).

Sedation score and GCMPS-SF were also significantly negatively correlated (Spearman’s rho −0.363, *p* = 0.007).

A correlation between PPF and GCMPS-SF was not indicated based on the significance (Spearman’s rho 0.248, *p* = 0.068).

### 3.6. Administration of Other Drugs

Most dogs in both groups received anti-inflammatory medications, primarily corticosteroids or NSAIDs. In the CCTM group, 25.9% (7/27) of dogs received NSAIDs, 59.3% (16/27) received corticosteroids, and 14.8% (4/27) did not receive any anti-inflammatory medication. Similarly, in the TLM group, 32.1% (9/28) of dogs received NSAIDs, 53.6% (15/28) received corticosteroids, and 14.3% (4/28) received no anti-inflammatory medication.

Administration of gabapentinoids was limited in both groups. In the CCTM group, 22.2% (6/27) of dogs received gabapentinoid treatment, whereas 77.8% (21/27) did not. In the TLM group, only 3.6% (1/28) of dogs received gabapentinoids, while 96.4% (27/28) did not receive gabapentinoid medication.

The only opioid administered was tramadol, which was required in a small number of dogs. In the CCTM group, 11.1% (3/27) of dogs received tramadol, whereas 88.9% (24/27) were not treated with this medication. Similarly, in the TLM group, 10.7% (3/28) of dogs received tramadol, while 89.3% (25/28) received no tramadol treatment.

Due to small sample size only descriptive statistics were produced (Table A3).

## 4. Discussion

Dogs affected by IVDD commonly present for diagnostic imaging or surgical management, thereby requiring the administration of general anaesthesia. Often, in the absence of serious concurrent diseases, these patients fall into the ASA II category. The ASA II classification designates a systemic disturbance that is moderate but distinct, with minimal effect on the patient’s physiology [3], allowing the anesthetist to administer safely a variety of anaesthetic drugs. These include but are not limited to DEX and PPF. As with all α2 agonists, it exerts its hypnotic action through activation of central pre- and postsynaptic α2-receptors in the Locus Coeruleus and is a valuable sedative in several settings. Activation of α2 receptors leads to hyperpolarization via potassium ions influx leading to decreased neuronal firing as well as reduced norepinephrine release resulting in inhibited sympathetic outflow via reduction in activity of the ascending noradrenergic pathways [10,18,19,20]. Even though DEX is one of the most potent and reliable sedatives, its results depend greatly on the patient’s arousal level at the time of administration [5]. Beyond stress associated with the clinical environment, painful conditions can further contribute to heightened patient stress, which may consequently reduce the degree of sedation achieved with dexmedetomidine. In fact, Locus Coeruleus plays an important role in pain modulation. Its activation causes inhibitory modulation in case of acute pain. However, in case of chronic conditions, Locus Coeruleus facilitates the upregulation of pain, contributing to lasting pain [21]. In this clinical, study this was highlighted by the sedation scores achieved in every patient, which were likely affected by pain sensation leading to stress. Higher scores in the GCMPS-SF were accompanied by lowest sedation scores, specifically in the CCTM group. Although both cervical and thoracolumbar intervertebral disc disease can be associated with significant pain, dogs suffering from cervical IVDD in this study exhibited higher GCMPS-SF scores compared with those affected by thoracolumbar IVDD. Differences in pain severity between these two conditions have been previously documented [22]. The authors attributed this finding to the fact that, in our study, 32% (9/28) of dogs with thoracolumbar IVDD presented with nociceptive deficits. These dogs were classified as grade 5/5 according to the Modified Frankel Scoring (MFS) system, indicating severe neurological impairment with markedly diminished pain perception. As the depth of sedation is a critical determinant of the injectable anesthetic dose required for induction, this reduction in nociception likely influenced the amount of PPF needed for anesthetic induction.

Propofol is the most widely used intravenous agent. It can provide smooth intubation conditions and its effects are mostly mediated through its interaction with GABA receptors [10]. The usual dose of PPF required to intubate a patient can vary greatly by the level of their arousal. In fact, premedication holds a great role in reducing the consciousness level of the patient thus reducing the required PPF dose for anesthesia induction [23,24]. Propofol is a short-acting intravenous anaesthetic used in both human and veterinary medicine for the induction and maintenance of general anaesthesia, usually following premedication [16]. Its widespread use is attributed to the rapid onset of anaesthesia within 30–60 s and the smooth, rapid recovery it provides [11,12,16]. However, PPF is known to cause dose-dependent respiratory depression, with its administration commonly resulting in apnoea and a reduced ventilatory response to hypoxaemia and hypercapnia [16]. Therefore, the administration of PPF in dogs with respiratory compromise due to lesions of the cervical and thoracic spinal cord should be carefully controlled [15].

The precise dosing of PPF in patients with focal spinal cord lesions such as IVDD has not been fully established in the literature. The effect of PPF on respiratory function, and specifically on diaphragmatic function, has been investigated in both experimental and clinical studies [25,26,27,28,29]. In an experimental study conducted in rats, 24 h sedation with PPF was shown to induce similar diaphragmatic dysfunction in both mechanically ventilated animals and those breathing spontaneously. Specifically, PPF caused a reduction in diaphragmatic contractile force ranging between 31% and 34% across the evaluated groups [26].

Comparable findings have also been reported in clinical studies in humans undergoing PPF sedation, in which changes in diaphragmatic contractility were assessed using ultrasonography [28,29]. In the preliminary study by Ranieri et al. (2015), a reduction in diaphragmatic contractility was recorded in seven patients undergoing endoscopic procedures under deep PPF sedation administered via target-controlled infusion (TCI) [28]. These findings are further supported by the study by Rocco et al. (2017) [29], in which ultrasonographic evaluation demonstrated a significant decrease in diaphragmatic contractility in patients under deep PPF sedation during endoscopic procedures. Nevertheless, the reduction in diaphragmatic function was not accompanied by changes in arterial oxygen hemoglobin saturation in patients [29]. In another study involving pediatric patients undergoing mechanical ventilation, PPF administration was shown to reduce diaphragmatic electrical activity. However, this study did not include the co-administration of other anaesthetic or analgesic agents, which could have influenced the results [27].

A single bolus administration of PPF in adult patients appears to induce a reversible inhibition of diaphragmatic contractility, as assessed by cervical magnetic stimulation. Specifically, patients exhibited suppression of transdiaphragmatic values during the action of PPF; however, diaphragmatic contractility returned to near-normal levels after the drug’s effects had subsided [25].

The negative impact of prolonged PPF administration on diaphragmatic contractility and, consequently, on respiratory function becomes evident based on the above findings. This is an important consideration in patients with respiratory compromise secondary to focal myelopathy, and may also be relevant to the dogs included in the present study, which presented with IVDD-related focal spinal cord disease. Nevertheless, single-bolus administration of PPF, as used for anaesthetic induction and endotracheal intubation, does not appear to impair diaphragmatic contractility, making it a particularly useful and safe agent in clinical practice [25].

Ensuring the safety of the anaesthetic protocol is of utmost importance, especially given the apparent increased PPF requirement in CCTM patients. Although the GCMPS-SF scores could not be directly correlated with PPF doses, dogs with higher GCMPS-SF scores, as mentioned above, exhibited lower SS. This reduced level of sedation may have contributed to increased PPF requirements for endotracheal intubation in dogs in the CCTM group. In the present clinical study, DEX 180 μg m^−2^ was administered as part of the premedication protocol. Interaction between these two anesthetic agents has been studied and it was indeed shown that DEX exerts a PPF-sparing effect in patients [30,31]. Apart from the sedative effect of premedication on SS reduction, the localization of IVDD may also have contributed to differences in sedation scores. In the present study, dogs in the CCTM group exhibited lower sedation scores, with a median value of 6/17, compared with dogs in the TLM group, which showed higher sedation scores with a median value of 7.5/17. The authors of the present study suggest that this finding may be associated with the intubation technique. In human medicine the intubation of patients with cervical myelopathies (mostly caused by trauma) has been adequately discussed. More specifically, to prevent additional spinal damage, these patients need to be handled with more delicacy. Spine stabilization can be achieved through different intubation techniques (fiber-optic guided intubation). The technique of intubation used and the distinctive clinical characteristics of the dogs with cervical myelopathy represent an aspect that warrants further clarification. Dogs included in this study were intubated in lateral recumbency, while an assistant was holding the head in a higher position, providing support to the neck with his dominant hand. Painful stimuli from these manipulations may result in sympathotonia and an increase in awareness in the CCTM group and may therefore explain the higher PPF requirements observed in these patients compared with the TLM group, in which the intubation procedure does not involve manipulation of the affected and more distant painful region. Determining the optimal induction dose in those patients remains challenging, and there is currently insufficient evidence to define standardized PPF dosing guidelines for dogs with cervical or thoracolumbar IVDD.

Exploring any potential relationship between PPF requirements and different intubation techniques, such as fiberoptic intubation compared with the conventional approach, particularly in dogs with cervical IVDD, would be of considerable clinical interest. In addition, assessing the association between neurological severity and PPF requirements in dogs with IVDD could provide valuable insight, as these parameters can be used by clinicians in routine veterinary practice.

The first limitation of this study is its retrospective design. Additionally, pain assessment relied solely on a single scale, the GCMPS SF. Although this is a validated tool for pain evaluation, it may be influenced by the animal’s temperament, potentially affecting the accuracy of the results. The inclusion of more objective measures, such as physiological indicators of nociception, as well as biochemical or neurophysiological markers, would have strengthened the assessment. Another limitation is the relatively small sample size, which prevented subgroup analyses. Specifically, we were unable to compare animals with different types of intervertebral disc disease, such as Hansen type I versus Hansen type II. These conditions may differ in terms of pain perception and anesthetic requirements, potentially influencing the outcomes observed in each group. Finally, we could not ensure uniformity between groups in order to compare the different co-administered drugs in a standardized way.

Further studies are warranted to confirm and expand upon the findings of this study and the factors influencing PPF dose requirements in dogs with IVDD.

## 5. Conclusions

According to the findings of this study, dogs with cervical compressive myelopathy due to IVDD require significantly higher doses of PPF for anaesthetic induction compared with dogs affected by IVDD-related thoracolumbar myelopathy.

This increased requirement correlates positively with lower pre-anaesthetic sedation, as assessed by the sedation score, suggesting that the level of sedation may influence anesthetic needs. These results highlight the importance of considering both the location of spinal pathology and sedation level when planning anesthetic protocols including PPF in dogs with IVDD. Further research with larger populations is warranted to validate these findings and to explore potential strategies for optimizing anesthetic management in dogs presented with IVDD.

## Data Availability

The datasets generated and analysed during the study are available from the corresponding author upon request.

## Abbreviations

The following abbreviations are used in this manuscript:

PPF	Propofol
IVDD	Intervertebral disc disease
DEX	Dexmedetomidine
CCTM	Cervical–cervicothoracic myelopathy
TLM	Thoracolumbar myelopathy
GCMPS-SF	Glasgow Composite Measure Pain Scale-Short Form
SS	Sedation Score
NSAIDs	Nonsteroidal anti-inflammatory drugs
MFS	Modified Frankel Scoring
GABA	Gamma-Aminobutyric Acid
SD	Standard deviation

## Appendix A

**Table A1:** Demographics.

Patient Number	Age (Years)	Sex (Male/Female- Neutered/Spayed)	Weight (kg)	Breed	Diagnosis (Hansen Type I/II)
CCTM 1	3.0	Male	11.0	French Bulldog	IVDD Type I C3–C4
CCTM 2	3.0	Male Neutered	13.0	French Bulldog	IVDD Type I C4–C5
CCTM 3	6.5	Male	9.4	Mongrel	IVDD Type I C3–C4
CCTM 4	5.0	Female Spayed	13.5	Cocker Spaniel	IVDD Type I C3–C4
CCTM 5	3.5	Female Spayed	9.0	French Bulldog	IVDD Type I C3–C4
					
CCTM 6	10.0	Male	13.5	Cocker Spaniel	IVDD Type II C3–C4
CCTM 7	8.0	Male	19.0	Greek Harehound	IVDD Type I C2–C3
CCTM 8	5.0	Male	12.0	French Bulldog	IVDD Type I C3–C4
CCTM 9	8.0	Female Spayed	10.8	French Bulldog	IVDD Type I C2–C3
CCTM 10	7.0	Female Spayed	12.0	Cocker Spaniel	IVDD Type I C2–C3
CCTM 11	6.0	Female	5.4	Mongrel	IVDD Type I C3–C4
CCTM 12	7.0	Male	4.0	Chihuahua	IVDD Type I C2–C3
CCTM 13	10.0	Male	8.4	Pincher	IVDD Type II C3–C4
CCTM 14	5.5	Female Spayed	11.0	French Bulldog	IVDD Type I C3–C4
CCTM 15	8.0	Female Spayed	7.0	Mongrel	IVDD Type I C2–C3
CCTM 16	9.0	Male Neutered	24.5	Cocker Spaniel	IVDD Type I C6–C7
CCTM 17	7.0	Male Neutered	14.3	Mongrel	IVDD Type I C3–C4
CCTM 18	7.0	Female	5.0	Pincher	IVDD Type I C4–C5
CCTM 19	4.0	Female Spayed	6.7	Mongrel	IVDD Type I C2–C3
CCTM 20	8.5	Male Neutered	35.0	Labrador Retriever	IVDD Type II C5–C6
CCTM 21	10.0	Male Neutered	9.0	Mongrel	IVDD Type I C5–C6
CCTM 22	10.0	Male Neutered	7.3	Mongrel	IVDD Type I C3–C4
CCTM 23	6.0	Male Neutered	11.8	French Bulldog	IVDD Type I C3–C4
CCTM 24	8.0	Male Neutered	15.0	Beagle	IVDD Type II C2–C3
CCTM 25	4.0	Female Spayed	10.5	French Bulldog	IVDD Type I C2–C3
CCTM 26	3.0	Female Spayed	10.3	French Bulldog	IVDD Type I C2–C3
CCTM 27	10.0	Male Neutered	28.0	GSD	IVDD Type II C6–C7
TLM 1	9.5	Male	9.0	Pincher	IVDD Type II T12–T13
TLM 2	8.5	Female	6.8	Maltese	IVDD Type I L2–L3
TLM 3	5.0	Female Spayed	6.7	Mongrel	IVDD Type I T13–L1
TLM 4	6.0	Female Spayed	17.0	Mongrel	IVDD Type I L2–L3
TLM 5	6.0	Male	8.0	Pekingese	IVDD Type II L2–L3
TLM 6	7.0	Male	6.5	West Highland Terrier	IVDD Type I L1–L2
TLM 7	5.5	Female Spayed	18.0	Mongrel	IVDD Type I T11–T12
TLM 8	3.5	Female Spayed	9.2	French Bulldog	IVDD Type I T12–T13
TLM 9	3.5	Female Spayed	7.4	Mongrel	IVDD Type I L2–L3
TLM 10	4.0	Female Spayed	14.6	French Bulldog	IVDD Type I L1–L2
TLM 11	2.0	Male	10.8	French Bulldog	IVDD Type I L2–L3
TLM 12	8.0	Female Spayed	23.0	Pit Bull	IVDD Type I T12–T13
TLM 13	6.0	Female Spayed	7.0	Pekingese	IVDD Type I T12–T13
TLM 14	2.0	Male	10.8	French Bulldog	IVDD Type I L2–L3
TLM 15	5.0	Male	25.8	Mongrel	IVDD Type I L1–L2
TLM 16	7.0	Male Neutered	6.0	Pekingese	IVDD Type I T12–T13
TLM 17	6.0	Male Neutered	7.0	Jack Russel	IVDD Type I T12–T13
TLM 18	10.0	Male Neutered	8.8	Mongrel	IVDD Type I T12–T13
TLM 19	3.5	Male Neutered	10.0	French Bulldog	IVDD Type I T12–T13
TLM 20	7.0	Male Neutered	8.0	Mongrel	IVDD Type I L2–L3
TLM 21	7.0	Male Neutered	11.6	Mongrel	IVDD Type I L1–L2
TLM 22	5.0	Female Spayed	6.6	Maltese	IVDD Type I L2–L3
TLM 23	4.0	Male Neutered	13.2	Mongrel	IVDD Type I T11–T12
TLM 24	3.0	Male Neutered	7.0	Jack Russel	IVDD Type I T12–T13
TLM 25	9.0	Male	7.5	Maltese	IVDD Type I T13–L1
TLM 26	10.0	Male	10.0	Pug	IVDD Type I T11–T12
TLM 27	9.0	Female Spayed	9.0	Mongrel	IVDD Type I L2–L3
TLM 28	8.0	Male Neutered	5.5	Maltese	IVDD Type I L1–L2

**Table A2:** Summary of patient characteristics, including diagnosis (Hansen type I/II), MFS score, sedation score, GCMPS-SF score and fractional values, and PPF dose (mg kg^−1^).

Patient Number	Diagnosis (Hansen Type I/II)	MFS Score	Sedation Score/17	GCMPS-SF	Fractions GCMPS-SF	Ppf Dose (mg kg^−1^)
CCTM 1	IVDD Type I C3–C4	2/5	6	10/24	0.42	3
CCTM 2	IVDD Type I C4–C5	1/5	4	14/24	0.58	4
CCTM 3	IVDD Type I C3–C4	1/5	5	16/24	0.67	2.5
CCTM 4	IVDD Type I C3–C4	2/5	7	12/24	0.50	2
CCTM 5	IVDD Type I C3–C4	2/5	6	17/24	0.71	3
CCTM 6	IVDD Type II C3–C4	1/5	7	15/24	0.63	3
CCTM 7	IVDD Type I C2–C3	4/5	5	12/20	0.60	4
CCTM 8	IVDD Type I C3–C4	3/5	4	15/24	0.63	2
CCTM 9	IVDD Type I C2–C3	4/5	3	12/24	0.50	2
CCTM 10	IVDD Type I C2–C3	3/5	4	14/24	0.58	1.5
CCTM 11	IVDD Type I C3–C4	3/5	3	16/24	0.67	3
CCTM 12	IVDD Type I C2–C3	4/5	5	14/24	0.58	2.5
CCTM 13	IVDD Type II C3–C4	3/5	6	7/24	0.29	3
CCTM 14	IVDD Type I C3–C4	4/5	5	18/24	0.75	6
CCTM 15	IVDD Type I C2–C3	2/5	4	13/24	0.54	4
CCTM 16	IVDD Type I C6–C7	2/5	8	5/24	0.21	2.5
CCTM 17	IVDD Type I C3–C4	4/5	9	16/24	0.67	3
CCTM 18	IVDD Type I C4–C5	3/5	7	5/24	0.21	3
CCTM 19	IVDD Type I C2–C3	4/5	7	18/24	0.75	4
CCTM 20	IVDD Type II C5–C6	3/5	5	15/24	0.63	5
CCTM 21	IVDD Type I C5–C6	1/5	10	5/24	0.21	2
CCTM 22	IVDD Type I C3–C4	4/5	7	7/20	0.29	4
CCTM 23	IVDD Type I C3–C4	2/5	7	13/24	0.54	3
CCTM 24	IVDD Type II C2–C3	3/5	10	7/24	0.29	3
CCTM 25	IVDD Type I C2–C3	1/5	5	10/24	0.42	3
CCTM 26	IVDD Type I C2–C3	2/5	2	0/24	0.00	7
CCTM 27	IVDD Type II C6–C7	3/5	7	0/24	0.00	2
TLM 1	IVDD Type II T12–T13	3/5	7	5/24	0.21	3
TLM 2	IVDD Type I L2–L3	1/5	10	0/24	0.00	2
TLM 3	IVDD Type I T13–L1	4/5	6	10/20	0.50	2
TLM 4	IVDD Type I L2–L3	5/5	7	7/20	0.35	2
TLM 5	IVDD Type II L2–L3	3/5	7	5/20	0.25	1.5
TLM 6	IVDD Type I L1–L2	4/5	8	7/20	0.35	1.5
TLM 7	IVDD Type I T11–T12	2/5	7	5/24	0.21	1.5
TLM 8	IVDD Type I T12–T13	5/5	8	7/24	0.29	1
TLM 9	IVDD Type I L2–L3	3/5	5	8/24	0.33	1
TLM 10	IVDD Type I L1–L2	5/5	2	12/24	0.50	1.5
TLM 11	IVDD Type I L2–L3	3/5	2	4/24	0.17	2
TLM 12	IVDD Type I T12–T13	5/5	4	7/20	0.35	2
TLM 13	IVDD Type I T12–T13	5/5	10	8/20	0.40	1.5
TLM 14	IVDD Type I L2–L3	3/5	6	10/24	0.42	1.5
TLM 15	IVDD Type I L1–L2	3/5	9	12/24	0.50	1.5
TLM 16	IVDD Type I T12–T13	4/5	8	12/20	0.60	2
TLM 17	IVDD Type I T12–T13	5/5	6	14/20	0.70	2
TLM 18	IVDD Type I T12–T13	5/5	8	5/20	0.25	2
TLM 19	IVDD Type I L2–L3	3/5	8	10/24	0.42	1.5
TLM 20	IVDD Type I L1–L2	3/5	12	14/24	0.58	2
TLM 21	IVDD Type I L2–L3	3/5	5	2/20	0.10	4
TLM 22	IVDD Type I T11–T12	5/5	10	2/20	0.10	2
TLM 23	IVDD Type I T12–T13	4/5	7	7/24	0.29	3
TLM 24	IVDD Type I T13–L1	4/5	3	10/20	0.50	2.5
TLM 25	IVDD Type I T11–T12	2/5	12	2/24	0.08	1
TLM 26	IVDD Type I L2–L3	2/5	10	0/24	0.00	2
TLM 27	IVDD Type I L1–L2	3/5	12	5/24	0.21	2
TLM 28	IVDD Type I T12–T13	5/5	9	2/20	0.10	2.5

**Table A3:** Patient number, diagnosis, and co-administered analgesic/anti-inflammatory medications, gabapentinoid and tramadol.

Patient Number	Diagnosis	Anti-Inflammatory Corticosteroids/NSAIDs/None	Gabapentinoids (YES/NO)	Tramadol (YES/NO)
CCTM 1	IVDD Type I C3–C4	Corticosteroids	Ν	Ν
CCTM 2	IVDD Type I C4–C5	NSAIDs	N	N
CCTM 3	IVDD Type I C3–C4	Corticosteroids	N	N
CCTM 4	IVDD Type I C3–C4	Corticosteroids	N	N
CCTM 5	IVDD Type I C3–C4	Corticosteroids	Y	Y
CCTM 6	IVDD Type II C3–C4	NSAIDs	Ν	Ν
CCTM 7	IVDD Type I C2–C3	Corticosteroids	N	N
CCTM 8	IVDD Type I C3–C4	Corticosteroids	N	N
CCTM 9	IVDD Type I C2–C3	Corticosteroids	N	N
CCTM 10	IVDD Type I C2–C3	Corticosteroids	Y	N
CCTM 11	IVDD Type I C3–C4	Corticosteroids	Y	N
CCTM 12	IVDD Type I C2–C3	Corticosteroids	Y	Y
CCTM 13	IVDD Type II C3–C4	NSAIDs	N	N
CCTM 14	IVDD Type I C3–C4	Corticosteroids	Y	Y
CCTM 15	IVDD Type I C2–C3	NSAIDs	N	N
CCTM 16	IVDD Type I C6–C7	NSAIDs	N	N
CCTM 17	IVDD Type I C3–C4	NSAIDs	N	N
CCTM 18	IVDD Type I C4–C5	NSAIDs	N	N
CCTM 19	IVDD Type I C2–C3	Corticosteroids	N	N
CCTM 20	IVDD Type II C5–C6	Corticosteroids	N	N
CCTM 21	IVDD Type I C5–C6	none	N	N
CCTM 22	IVDD Type I C3–C4	Corticosteroids	N	N
CCTM 23	IVDD Type I C3–C4	Corticosteroids	Y	N
CCTM 24	IVDD Type II C2–C3	none	Ν	Ν
CCTM 25	IVDD Type I C2–C3	Corticosteroids	Ν	Ν
CCTM 26	IVDD Type I C2–C3	none	Ν	Ν
CCTM 27	IVDD Type II C6–C7	none	N	N
TLM 1	IVDD Type II T12–T13	Corticosteroids	N	N
TLM 2	IVDD Type I L2–L3	Corticosteroids	N	N
TLM 3	IVDD Type I T13–L1	NSAIDs	N	N
TLM 4	IVDD Type I L2–L3	NSAIDs	N	N
TLM 5	IVDD Type II L2–L3	NSAIDs	N	N
TLM 6	IVDD Type I L1–L2	Corticosteroids	N	Y
TLM 7	IVDD Type I T11–T12	NSAIDs	N	N
TLM 8	IVDD Type I T12–T13	Corticosteroids	N	N
TLM 9	IVDD Type I L2–L3	NSAIDs	N	N
TLM 10	IVDD Type I L1–L2	Corticosteroids	N	N
TLM 11	IVDD Type I L2–L3	NSAIDs	N	N
TLM 12	IVDD Type I T12–T13	Corticosteroids	N	N
TLM 13	IVDD Type I T12–T13	Corticosteroids	N	N
TLM 14	IVDD Type I L2–L3	Corticosteroids	N	N
TLM 15	IVDD Type I L1–L2	Corticosteroids	N	Y
TLM 16	IVDD Type I T12–T13	NSAIDs	N	Υ
TLM 17	IVDD Type I T12–T13	Corticosteroids	N	N
TLM 18	IVDD Type I T12–T13	none	N	N
TLM 19	IVDD Type I L2–L3	Corticosteroids	Y	N
TLM 20	IVDD Type I L1–L2	Corticosteroids	Ν	Ν
TLM 21	IVDD Type I L2–L3	Corticosteroids	Ν	Ν
TLM 22	IVDD Type I T11–T12	Corticosteroids	Ν	Ν
TLM 23	IVDD Type I T12–T13	Corticosteroids	N	N
TLM 24	IVDD Type I T13–L1	NSAIDs	N	N
TLM 25	IVDD Type I T11–T12	none	N	N
TLM 26	IVDD Type I L2–L3	none	Ν	Ν
TLM 27	IVDD Type I L1–L2	none	Ν	Ν
TLM 28	IVDD Type I T12–T13	NSAIDs	Ν	Ν

## References

[1] Dewey C.W., da Costa R.C., Dewey C.W., da Costa R.C. (2016). Myelopathies: Disorders of the Spinal Cord. Practical Guide to Canine and Feline Neurology.

[2] da Costa R.C., De Decker S., Lewis M.J., Volk H. (2020). Diagnostic Imaging in Intervertebral Disc Disease. Front. Vet. Sci..

[3] Portier K., Ida K.K. (2018). The ASA Physical Status Classification: What Is the Evidence for Recommending Its Use in Veterinary Anesthesia?—A Systematic Review. Front. Vet. Sci..

[4] Duke-Novakovski T., de Vries M., Seymour C.M. (2016). BSAVA Manual of Canine and Feline Anaesthesia and Analgesia.

[5] Grimm K.A., Lamont L.A., Tranquilli W.J., Greene S.A., Robertson S.A. (2015). Sedatives and Tranquilizers. Veterinary Anesthesia and Analgesia: The Fifth Edition of Lumb and Jones.

[6] Lin P.-J., Lin L.-S., Chung C.-S. (2024). Intranasal Dexmedetomidine as Premedication for Magnetic Resonance Imaging Examinations in Dogs with Neurological Disorders Mitigates Hypotension and Hypothermia. J. Am. Vet. Med. Assoc..

[7] Smith C.K., Seddighi R., Cox S.K., Sun X., Knych H.K., Doherty T.J. (2017). Effect of Dexmedetomidine on the Minimum Infusion Rate of Propofol Preventing Movement in Dogs. Vet. Anaesth. Analg..

[8] Beier S.L., de Lima M.P.A., de Sousa F.G., Silva R.A., Fagundes N., Bovi M.F., Tôrres R.C.S. (2023). Comparison of Two Sedation Protocols for Diagnostic Radiography in Dogs with Hip Dysplasia. Vet. Anim. Sci..

[9] Ko J.C., Murillo C., Weil A.B., Kreuzer M., Moore G.E. (2024). Dexmedetomidine Sedation in Dogs: Impact on Electroencephalography, Behavior, Analgesia, and Antagonism with Atipamezole. Vet. Sci..

[10] Hemmings H., Egan T. (2018). Pharmacology and Physiology for Anesthesia: Foundations and Clinical Application.

[11] Walsh C.T. (2018). Propofol: Milk of Amnesia. Cell.

[12] Bollucuoglu K., Hanci V., Yurtlu S., Okyay D., Ayoglu H., Turan I.O. (2013). Comparison of Propofol-Dexmedetomidine, Thiopental-Dexmedetomidine and Etomidate-Dexmedetomidine Combinations’ Effects on Tracheal Intubation Conditions. Bratisl. Med. J..

[13] Clarke K.W., England G.C.W. (1989). Medetomidine, a New Sedative-Analgesic for Use in the Dog and Its Reversal with Atipamezole. J. Small Anim. Pract..

[14] Jensen T.S., Finnerup N.B. (2014). Allodynia and hyperalgesia in neuropathic pain: Clinical manifestations and mechanisms. Lancet Neurol..

[15] Sarpekidou E., Pavlidou K., Savvas I., Polizopoulou Z., Kazakos G. (2025). Differences in Transdiaphragmatic Pressure of Dogs Suffering from Cervical or Thoracolumbar Myelopathy Anaesthetised with Isoflurane. Animals.

[16] Berry S.H., Grimm K.A., Tranquilli W.J. (2015). Injectable Anesthetics. Veterinary Anesthesia and Analgesia: The Fifth Edition of Lumb and Jones.

[17] Vainio O., Vähä-Vähe T., Palmu L. (1989). Sedative and Analgesic Effects of Medetomidine in Dogs. J. Vet. Pharmacol. Ther..

[18] Coursin D.B., Maccioli G.A. (2001). Dexmedetomidine. Curr. Opin. Crit. Care.

[19] Giovannitti J.A., Thoms S.M., Crawford J.J. (2015). Alpha-2 Adrenergic Receptor Agonists: A Review of Current Clinical Applications. Anesth. Prog..

[20] Carollo D.S., Nossaman B.D., Ramadhyani U. (2008). Dexmedetomidine: A Review of Clinical Applications. Curr. Opin. Anaesthesiol..

[21] Suárez-Pereira I., Llorca-Torralba M., Bravo L., Camarena-Delgado C., Soriano-Mas C., Berrocoso E. (2022). The Role of the Locus Coeruleus in Pain and Associated Stress-Related Disorders. Biol. Psychiatry.

[22] Züger L., Fadda A., Oevermann A., Forterre F., Vandevelde M., Henke D. (2018). Differences in Epidural Pathology between Cervical and Thoracolumbar Intervertebral Disk Extrusions in Dogs. J. Vet. Intern. Med..

[23] Goyagi T., Tanaka M., Nishikawa T. (2000). Oral Clonidine Premedication Reduces Propofol Requirement for Laryngeal Mask Airway Insertion. Can. J. Anaesth..

[24] Bovill J.G., Olmos M., Ballester J.A., Vidarte M.A., Elizalde J.L., Escobar A. (2000). The Combined Effect of Age and Premedication on Propofol Requirements for Induction by Target-Controlled Infusion. Anesthesiology.

[25] Zhang X.J., Yu G., Wen X.H., Lin Z.C., Yang F.Q., Zheng Z.G., Chen R.C., Zhong N.S. (2009). Effect of Propofol on Twitch Diaphragmatic Pressure Evoked by Cervical Magnetic Stimulation in Patients. Br. J. Anaesth..

[26] Bruells C.S., Maes K., Rossaint R., Thomas D., Cielen N., Bergs I., Bleilevens C., Weis J., Gayan-Ramirez G. (2014). Sedation Using Propofol Induces Similar Diaphragm Dysfunction and Atrophy during Spontaneous Breathing and Mechanical Ventilation in Rats. Anesthesiology.

[27] Amigoni A., Rizzi G., Divisic A., Brugnaro L., Conti G., Pettenazzo A. (2015). Effects of Propofol on Diaphragmatic Electrical Activity in Mechanically Ventilated Pediatric Patients. Intensive Care Med..

[28] Ranieri G., Maggi L., Belsito F., Rocco M., De Blasi R.A. (2015). Propofol Sedation Reduces Contraction and Motion of Diaphragm in Humans. Crit. Care.

[29] Rocco M., Maggi L., Ranieri G., Ferrari G., Gregoretti C., Conti G., De Blasi R.A. (2017). Propofol Sedation Reduces Diaphragm Activity in Spontaneously Breathing Patients. Minerva Anestesiol..

[30] Sanches M.C., Bessi W.H., Rusch E., Schaffhausser M.B., Cassoli A.A., Freitas S.H., Gehrcke M.I., Carregaro A.B. (2022). Cardiopulmonary and Propofol-Sparing Effects of Dexmedetomidine in Total Intravenous Anesthesia in Cats Undergoing Ovariohysterectomy. J. Feline Med. Surg..

[31] Li X., Ye Z., Cui M., Hu A., Li X., Chen Q., Zhao G., Ye F. (2023). Dexmedetomidine Decreases the 50% Effective Dose (ED50) of Intravenous Propofol Required to Prevent Tracheal Intubation Response in Beagles. J. Am. Vet. Med. Assoc..

